# Carvacrol and Thymol Hybrids: Potential Anticancer and Antibacterial Therapeutics

**DOI:** 10.3390/molecules29102277

**Published:** 2024-05-12

**Authors:** Sijongesonke Peter, Namhla Sotondoshe, Blessing A. Aderibigbe

**Affiliations:** Department of Chemistry, University of Fort Hare, Alice 5700, South Africa; 202000975@ufh.ac.za

**Keywords:** carvacrol, thymol, hybrid compounds, therapeutics, anticancer, antibacterial

## Abstract

Cancer is ranked among lethal diseases globally, and the increasing number of cancer cases and deaths results from limited access to effective therapeutics. The use of plant-based medicine has been gaining interest from several researchers. Carvacrol and its isomeric compound, thymol, are plant-based extracts that possess several biological activities, such as antimalarial, anticancer, antifungal, and antibacterial. However, their efficacy is compromised by their poor bioavailability. Thus, medicinal scientists have explored the synthesis of hybrid compounds containing their pharmacophores to enhance their therapeutic efficacy and improve their bioavailability. Hence, this review is a comprehensive report on hybrid compounds containing carvacrol and its isomer, thymol, with potent anticancer and antibacterial agents reported between 2020 and 2024. Furthermore, their structural activity relationship (SAR) and recommended future strategies to further enhance their therapeutic effects will be discussed.

## 1. Introduction

Several processes such as dysfunctional signalling transduction, the production of genomic instability, the evasion of immune destruction, metastasis and aberrant gene expression, and angiogenesis contribute to the development of uncontrolled cell growth known as cancer [1,2]. The lack of effective therapeutics for the treatment of cancer has been a great challenge to public health systems globally [1]. The complexity of cancer treatment is a major contributing factor to the significant progress made so far in the development of anticancer agents [1,3]. Due to the limitations associated with treatment strategies, such as chemotherapy, immunotherapy, and surgery, that are used to treat cancer, more than 10 million deaths were reported in 2020 [4,5,6]. More metastatic cancer cases were reported in 2020 because early cancer diagnosis was compromised by the emergence of COVID-19 [7,8,9].

Similarly, bacterial infections are also a major burden to the public health system globally. The issue of antibiotic resistance is a risk factor that compromises the efficacy of most antibacterial drugs [10,11,12]. More than 700,000 patients die yearly due to a lack of effective treatments, with an estimated 10 million lives being lost due to antibiotic-resistant pathogens each year. Furthermore, this has the potential to put a serious strain on the global economy, as it is estimated that trillions of dollars will be lost due to antibiotic resistance by 2050 [11,12]. There is an urgent need for effective therapeutics to be developed to treat cancer and bacterial infections.

**Figure 1:**
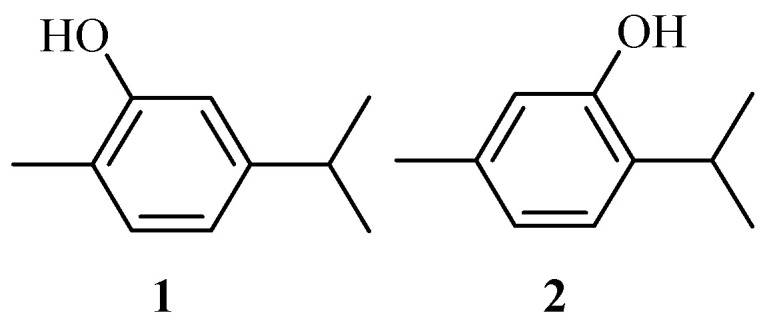
Chemical structures of carvacrol (**1**) and thymol (**2**).

Plant-based compounds such as thymol and carvacrol (Figure 1) are being explored for the development of new medicines with limited side effects [5,13,14,15]. Fifty percent of available drugs in the market are produced from natural products [13]. Therefore, the extraction and development of natural compounds are interesting areas of research. Carvacrol and its derivatives belong to the monoterpene class of natural products [4,16]. This class of natural compounds possesses antifungal, anticancer, antioxidant, antiparkinsonian, anti-anxiety, and antibacterial activities [4,5,13,17,18,19]. They have been used in the design of hybrid compounds.

The concept of hybridization is an effective and exciting strategy to develop new therapeutics [20,21]. It involves forming a single-entity drug from the combination of two or more pharmacophores. This strategy is characterized by promising advantages, such as increased patient compliance, multiple targets, reduced drug–drug interaction, etc. It is a promising drug design strategy that is effective in overcoming limitations such as drug resistance and drug–drug interactions, which are common with most chemotherapeutics [20,21]. Based on the aforementioned features of hybridization, several medicinal scientists have investigated the anticancer and antibacterial activity of carvacrol and thymol when hybridized with other pharmacophores. In this review, an update on carvacrol- and thymol-based hybrid compounds developed as potential antibacterial and anticancer therapeutic agents reported between 2020 and 2024 is presented.

## 2. Carvacrol and Thymol Synopsis

Carvacrol (**1**) and thymol (**2**) (Figure 1) are isomeric compounds from the monoterpenoid phenol family [22]. These essential oils are extracted from several groups of aromatic plants such as the Lamiaceae family, Thymus, Satureja, Origanum, Thymbra, Lippia pepperwort, Corydothymus, and Wild bergamot [23]. They are liquid phenolic monoterpenes that exist in the mentioned aromatic plants with boiling and melting points in the range of 236–237 ℃ and 3–4 ℃, respectively. They have also been synthesized by multiple biotechnological techniques. They are very soluble in diethyl ether, acetone, and ethanol but are not soluble in water [24,25].

**Figure 2:**
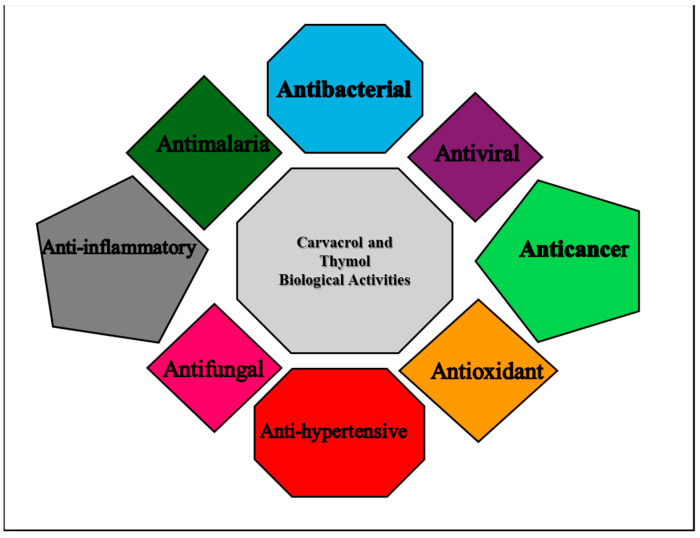
Biological activities of thymol and carvacrol.

These isomeric compounds have been used as preservative and biomedical applications, owing to their anaesthetic, antimicrobial, antifungal, antioxidant, anti-inflammatory, anticancer activities, etc. (Figure 2). Additionally, they have been used in perfumery and cosmetics [24,25,26,27,28]. They are effective in reducing the rate of food spoilage and pathogenic bacteria growth [25,26]. The presence of a free hydroxyl group and the phenol ring contributes to the antibacterial and antioxidant activities of carvacrol and thymol [29,30].

**Figure 3:**
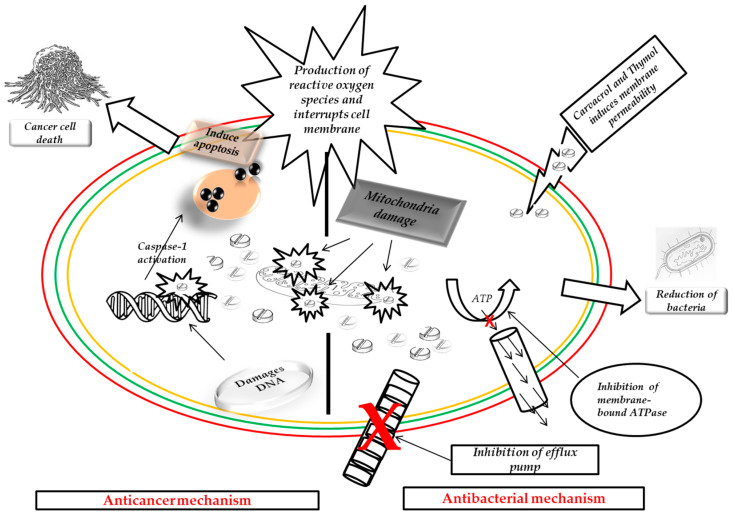
Carvacrol and thymol’s anticancer and antibacterial mechanisms of action.

The mechanism of action of these two aforementioned essential oils involves multi-targeting actions [31]. Therefore, their modes of action might depend on the pathogens and tumours targeted. The antibacterial action of these two isomeric compounds has been linked to their significant effects on the cytoplasmic membrane’s structural and functional characteristics (Figure 3). In essence, their mode of action is associated with the destruction of the bacteria cell membrane [29,32]. On the other hand, several studies have confirmed that the primary mechanism of action of carvacrol and thymol involves the decrease in cancer cell viability and their capability to induce apoptosis through both intrinsic and extrinsic routes (Table 1) [33,34,35,36]. Additionally, they produce more reactive oxygen species (ROS), which interrupt the DNA of cancer cells [33,34,35,36].

Carvacrol and thymol are regarded as safe compounds, and they possess several biological activities. Thus, their ability to simultaneously target several cells, pathogens, strains, etc., due to their vast mechanism of action is one of the advantages these two isomeric compounds when used in health applications [27,37,38,39,40,41,42]. However, their use is compromised by several limitations, such as drug resistance, toxicity in high doses, poor solubility in water, and poor drug delivery, which lead to poor bioavailability, low stability, and high hydrophobicity [24,41,42]. The use of high concentrations of these isomeric compounds results in mutagenicity and genotoxicity. Additionally, skin and eye irritation are side effects associated with carvacrol and thymol [24]. Thus, finding an alternative approach to improve their use in health applications is an interesting topic that needs to be addressed.

The hydroxyl functional group contributes to the antibacterial activity of the two phenolic compounds. In contrast, the anticancer structural activity relationship of these two compounds is still debatable [24,42]. These compounds have been applied in several therapeutic applications, including antibacterial and anticancer treatments. However, their efficacy in both combination therapy and monotherapy is compromised by some limitations, e.g., poor bioavailability, etc. [43,44,45]. Hence, better strategies to improve their therapeutic effect are a pressing need. Thus, the development of hybrid drugs using these two isomeric compounds may result in new therapeutic agents that can overcome their shortcomings [20]. Hybrid drugs are a cocktail of drugs developed through a combination of two or more drugs into a single drug molecule with reduced toxicity, dual targets, fewer side effects, and improved biological activities [20].

## 3. Carvacrol- and Thymol-Based Hybrid Compounds with Anticancer Activity

**Figure 4:**
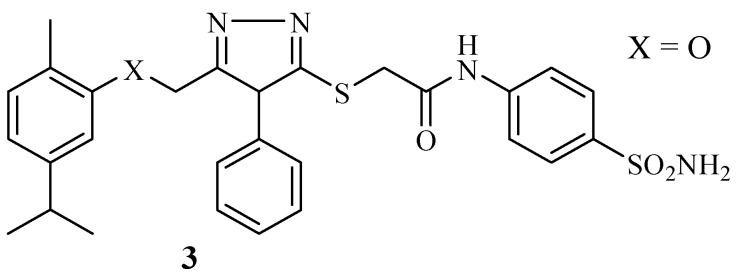
Chemical structure of the most active hybrid derivative **3** from the carvacrol generation synthesized by Demirbolat et al.

The development of novel therapeutics using plant-based molecules is one interesting area of research for medicinal scientists due to their biologically friendly properties and the non-toxicity of plant-based molecules to normal cells [18,46]. Thus, using them for the development of hybrid drug molecules can overcome their limitations and is a promising drug design strategy. Demirbolat et al. validated the hybrid synthetic approach through the synthesis of a series of carvacrol hybrid molecules (Figure 4) [1]. The hybrid compounds’ cytotoxic effect was tested on several cancer cells, including NIH/3T3, PC-3, MCF-7, K562, A549, and SH-SY5Y [1]. The findings displayed promising percentage proliferation inhibition rates which ranged between −39.03% and 40.62% against MCF-7 cancer cell lines. However, only compound **3** with a benzene ring and sulfonamide group on the triazole moiety displayed an IC_50_ value of 12.8 µM, which revealed a significant cytotoxic effect when compared to that of doxorubicin (49.05 µM). The rest of the synthesized molecules were found to be inactive with IC_50_ values greater than 100 µM against the MCF-7 cancer cell line. Furthermore, compound **3** promoted apoptosis in a dose-dependent manner. The toxicity studies from the in-silico studies of hybrid **3** showed that the compound exhibited no tumorigenic or mutagenic effects. This compound did not display an anticancer effect against PC-3, although its isomeric partner (thymol hybrid derivatives) from the previous study by the same group [47] displayed an IC_50_ of 5.96 µM against the same cancer cell in vitro. Hence, further studies are recommended [1]. The SAR of these hybrids synthesized by Demirbolat et al. displayed no consistent trend. However, the results of the percentage inhibition of proliferation depicted that the introduction of the benzene ring, sulfonamide group, and halogens to the triazole moiety influenced the anticancer activity of the compound. Therefore, further structural elucidation is paramount.

**Figure 5:**
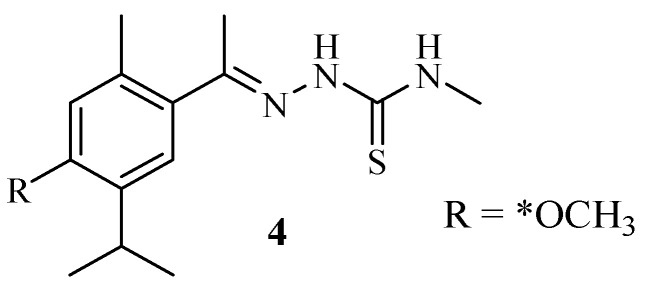
Chemical structure of hybrid **4** synthesized via a combination of thiosemicarbazone and thymol by Laamari et al.

Laamari et al. synthesized thymol hybrids and evaluated their anticancer effect on four different cancer cell lines (MCF-7, A-549, MDA-MB-231, and HT-1080) [9]. Most of the synthesized compounds displayed moderate levels of cytotoxicity in comparison with those of the reference drug, with IC_50_ values in the range of 7.10–50 μM. The cytotoxic effect of compound **4** (a combination of thiosemicarbazone and thymol) was significant when compared to that of other compounds in the series against almost all of the selected cancer cell lines with IC_50_ values between 7.10 and 19 μM. Specifically, against HT-1080 cancer cells, compound **4** (Figure 5) exhibited comparable anticancer activity to that of the control drug (6.21 μM) with IC_50_ values of 7.10 μM. The mode of action of the compound against A-549 and HT-1080 includes the induction of early and late apoptosis via cell cycle arrest in the G2/M-phase and caspase-3/7 activation. However, in vivo results were recommended to further validate the anticancer activity of this compound. Notably, replacing the hydrogen molecule with the methyl group on the side chain of the thiosemicarbazone improved the anticancer activity of this hybrid [9].

**Figure 6:**
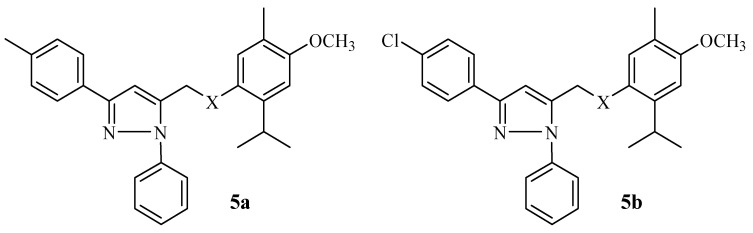
p-methoxythymol pyrazole hybrids **5a**–**b** synthesized by Laamari et al.

Laamari et al. further synthesized p-methoxy thymol pyrazole hybrids via 1,3-dipolar cycloaddition methods. Four different human cancer cells used in their previous study were used to test the anticancer effect of these hybrids [9], and the same control drug was used [18]. All the compounds displayed moderate anticancer effects with IC_50_ values ranging from 22.17 to 62.72 µM against all selected cancer cell lines [18]. The p-methoxy thymol pyrazole hybrids showed better cytotoxic effects when compared to other thymol pyrazole hybrids. The ether group on the thymol moiety influenced the improved anticancer activity. Hence, hybrids **5a** and **5b** (Figure 6) were regarded as the most active compounds against the A-549 and HT-1080 cell lines. Notably, the cytotoxicity IC_50_ values further improved in the range of 17.28–22.17 µM and 11.40–23.79 µM with an extended incubation period against the most sensitive cell line (A-549), respectively. Therefore, the anticancer effect of these hybrid drugs was time and structure dependent. However, further validation studies are recommended [18].

**Figure 7:**
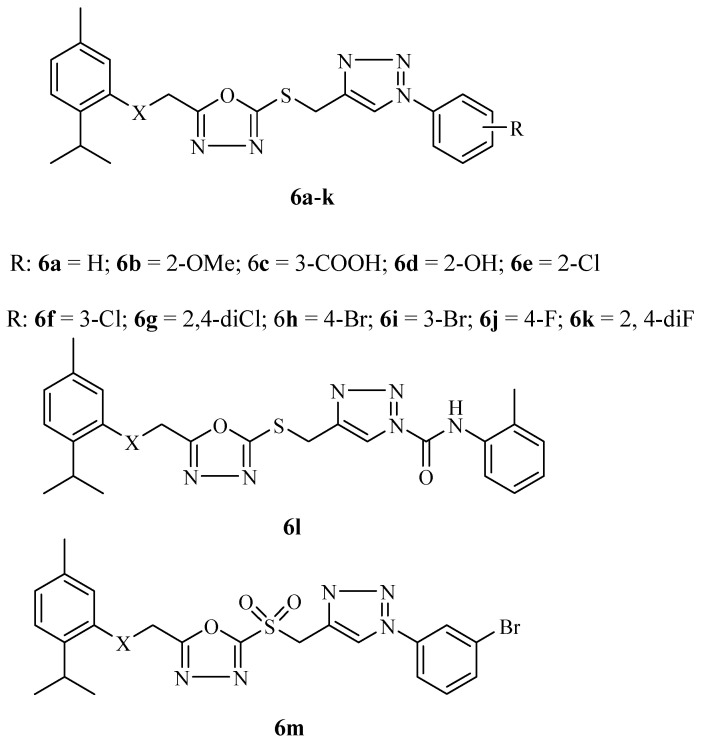
Chemical structure of hybrids **6a**–**m** synthesized by Almalki et al.

Almalki et al. synthesized thymol hybrid compounds after they displayed good druglikeness and pharmacokinetics properties in silico [48]. The synthesized hybrids **6a**–**m** (Figure 7) displayed significant antiproliferative results when compared to the reference drugs (doxorubicin (IC_50_ = 1.2 µM) and 5-fluorouracil (IC_50_ = 18.74 µM)) with no obvious trends when evaluated against HCT-116, MCF-7, and HepG2 cancer cell lines. The hybrids displayed selective anticancer effects on the cancer cell lines, with compounds **6b**–**f** being the most potent compounds against MCF-7 with IC_50_ values in the range of 1.1–4.9 µM. Hybrids **6b** and **6d** inhibited the growth of HepG2 cell lines with IC_50_ values of 1.8 µM and 1.4 µM, respectively. Against HCT-116 cell lines, only compound **6d** showed promising results with an IC_50_ value of 2.6 µM. These antiproliferative results were comparable to those of doxorubicin but were 15–20-fold more active than 5-fluorouracil [48]. The SAR of these hybrids revealed that the number and position of substituents influenced the anticancer effect [48]. The biological activity of the hybrids was not enhanced for the meta- and para-substituted hybrids when compared to the ortho-substituted hybrids with improved cytotoxicity. Increasing the number of substituents reduced their cytotoxic effects when compared to the monosubstituted hybrids. The introduction of a sulfone group also reduced the cytotoxic effect of the hybrids [48].

**Figure 8:**
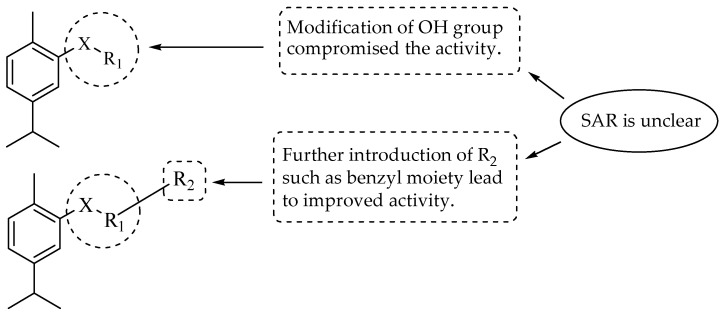
SAR of the carvacrol hybrids synthesized by Sisto et al.

Carvacrol hybrids (Figure 7) were reported by Sisto et al. [49]. In vitro cytotoxicity tests were performed on gastric adenocarcinoma cell lines. Among seventeen carvacrol hybrid derivatives, five showed a loss of activity, as their cell viability was inferior to that of carvacrol. All the hybrids exhibited poor activity compared to that of the control drug, 5-Fluorouracil. The SAR trend was unclear. However, a direct substitution to the hydroxyl functional group of carvacrol resulted in a significant loss of biological activity (Figure 8). The introduction of the benzyl moiety specifically with 3-CH_3_, 4-SO_2_CH_3_, 4-CF_3_, and 4-SOCH_3_ on the meta- and para-positions improved the biological activity of the hybrid compounds. In essence, the position of the substitution influenced the activity of these hybrid derivatives. Hence, further optimization was recommended [49].

**Figure 9:**
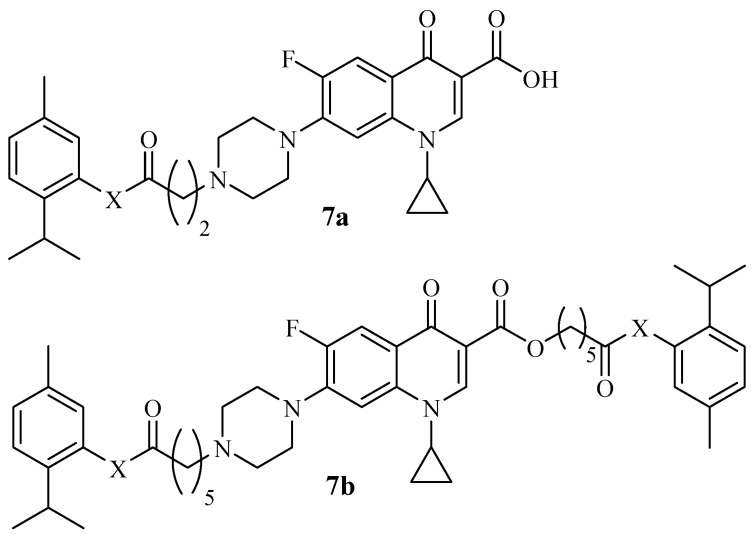
Chemical structures of thymol–ciprofloxacin hybrids **7a**–**b** synthesized by Szostek et al.

Szostek et al. synthesized thymol–ciprofloxacin hybrids (Figure 9), which were evaluated against four cancer and one normal human cell lines [50]. The cytotoxic effects of most of the hybrids were moderate against all of the cancer cell lines. Compound **7a**–**b** displayed a promising anticancer activity against the cancer cells with IC_50_ values less than 52 µM. No significant cytotoxic effect was visible on the normal human cell lines. The selective index (SI) values of hybrid **7a**–**b** were in the range of 1.9 to 3.4, revealing insignificant anticancer activity when compared to the control drug, doxorubicin, with SI values in the range of 0.14–1.11. However, unlike doxorubicin, which displayed cytotoxicity against all of the used cell lines, including normal cells, compounds **7a** and **7b** were cytotoxic towards only the cancer cell lines. There was no obvious SAR trend in these compounds. Therefore, these compounds are recommended for further studies [50].

**Figure 10:**
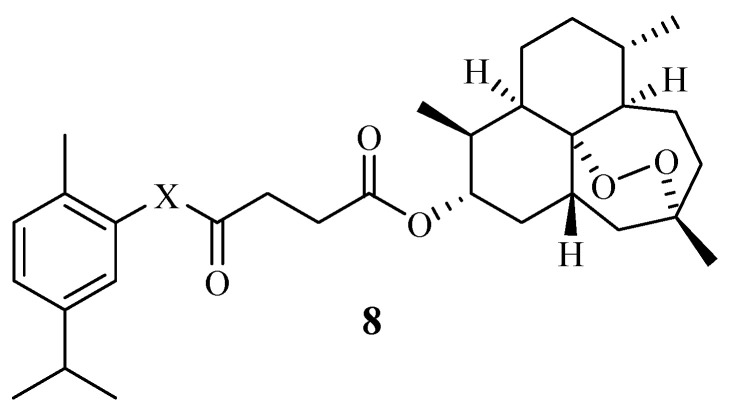
Hybrid **8** synthesized by Mbese et al.

Carvacrol hybrid compounds synthesized by Mbese et al. [51] were potent anticancer compounds with IC_50_ values between 0.47 and 16.57 µM. Specifically, compound **8** (Figure 10) displayed significant results against MCF-7 and MCF-12A with IC_50_ values of 0.47 and 0.75 µM, respectively. The improved anticancer activity was attributed to the incorporation of artesunate via an ester linker into the carvacrol moiety. Hence, hybridizing carvacrol with other anticancer pharmacophores is a promising approach. However, further studies are recommended [51].

**Figure 11:**
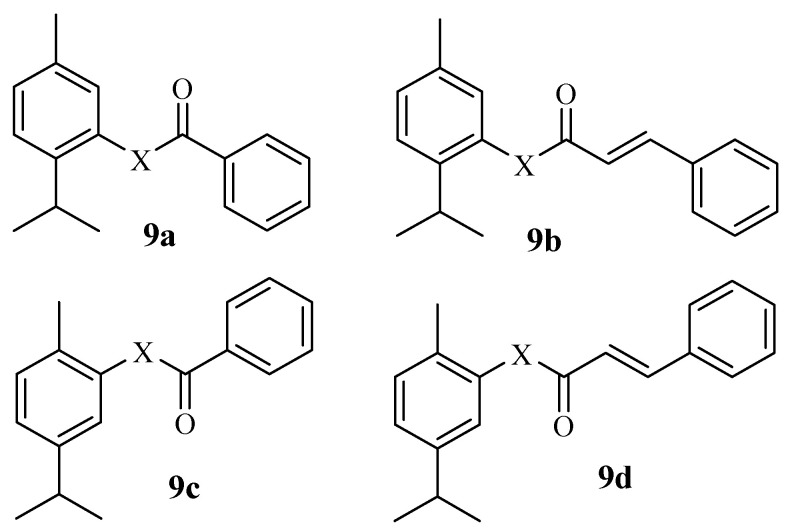
Hybrids **9a**–**d** synthesized by Valverde Sancho et al.

Valverde Sancho et al. synthesized hybrid compounds through the combination of carvacrol, eugenol, and cinnamic acid and tested their antibacterial and anticancer properties [52]. Among the synthesized compounds, carvacrol and thymol hybrids (**9a**–**d**) showed potent anticancer effects with LC_50_ values in the range of 50.39–71.95 µg/mL. Compound **9a** (Figure 11), which was synthesized through a combination of thymol and benzoic acid, was the most significant anticancer agent with an LC_50_ value of 50.39 µg/mL. Notably, a combination of Thyme vulgaris essential oils and Cinnamomum verum extracted compounds resulted in effective therapeutic agents [52]. The SAR of these hybrids did not follow a significant trend. However, the incorporation of cinnamic acid and benzoic acid moieties into thymol and carvacrol via ester linkers improved their anticancer activity [52].

**Figure 12:**
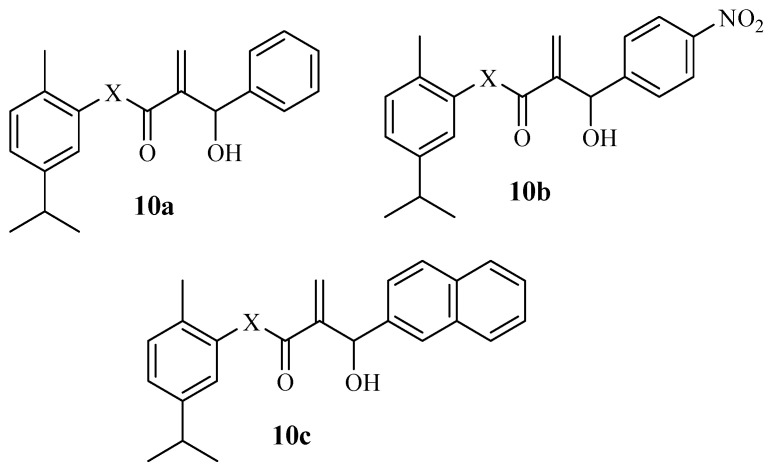
Carvacrol hybrids **10a**–**c** synthesized by Vasconcelos et al.

Vasconcelos et al. reported carvacrol derivatives with cytotoxic effects against SH-SY5Y and HEK-293 cancer cell lines [53]. Most of the hybrids exhibited an anticancer effect that was 10-fold more effective than carvacrol (IC_50_ = 374.1 µM) with IC_50_ values between 9.79 and 64.72 µM [53]. Notably, compounds **10a**–**c** (Figure 12) were the most active anticancer hybrids with a selective index of more than 3.0 compared to that of carvacrol (0.93), suggesting that the derivatives have more anticancer properties compared to the parent drug. Additionally, SAR indicated that the nitro position favouring para positions and the type of halogen present were influential on the anticancer effect of these compounds. Due to its promising anticancer effect, in vivo mechanistic studies and clinical trials were suggested for compound **10c** [53].

**Figure 13:**
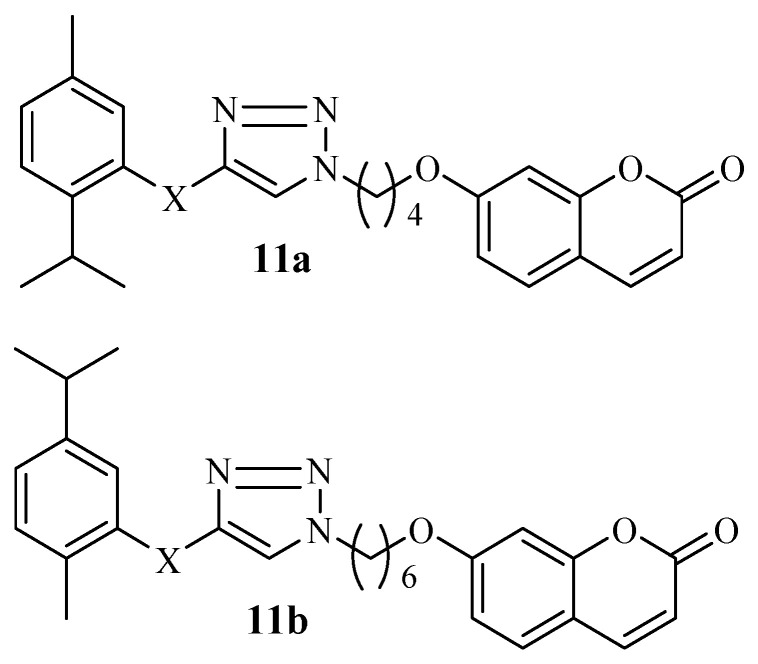
Thymol and carvacrol coumarin hybrids **11a**–**b**.

Interesting anticancer findings on two generations of coumarin–monoterpenes, including thymol and carvacrol moieties, were reported by Zengin et al. [54]. The cytotoxic effect of the hybrid compounds was studied using PC3, HT-29, HEK293T, and MCF-7 cell lines. The different linkers explored in these hybrids did not induce a significant trend in their anticancer activity. However, the hybridization of coumarin with monoterpenes is a promising approach to developing potent therapeutic agents. Thus, the hybrids were selective towards the cell lines, with thymol (**11a**) and carvacrol (**11b**) hybrids (Figure 13) displaying good anticancer activity after several evaluations, with **11a** exhibiting an IC_50_ value of 2.48 μM against MCF-7 and **11b** exhibiting values of 9.10, 9.40, and 12.01 μM against MCF-7, PC-3, and HT-29, respectively. The IC_50_ values of the compounds revealed promising anticancer activity, and they both induced apoptosis in MCF-7 and HT-29, respectively. Hence, further studies on compounds **11a** and **11b** are recommended [54].

**Figure 14:**
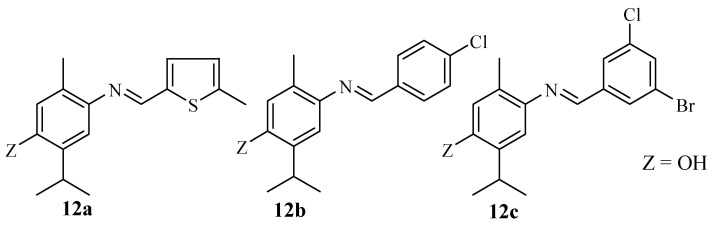
Chemical structure of thymol-based hybrid **12a**–**c** synthesized by Sahin et al.

Sahin et al. reported the anticancer activity of thymol hybrid compounds **12a**–**c** (Figure 14) [55]. The compounds were screened against several cancer cells. The hybrids **12a**–**c** were selective towards cell lines exhibiting IC_50_ values, revealing a superior anticancer activity to that of the control drug with some exceptions. Compound **12a** with the 5-methylthiophene group (IC_50_ value = 7.67 μM) and **12c** with the 3-bromo-5-chlorobenzylideneamino group (IC_50_ value = 12.39 μM) showed significant cytotoxic effects when compared to cisplatin (IC_50_ value = 16.27 and 19.16 μM) against PC3 and DLD-1 cancer cell lines, respectively. The structural modification displayed no cytotoxic influential trend in the hybrids’ anticancer effect [55]. Therefore, further studies on these hybrids are paramount.

**Figure 15:**
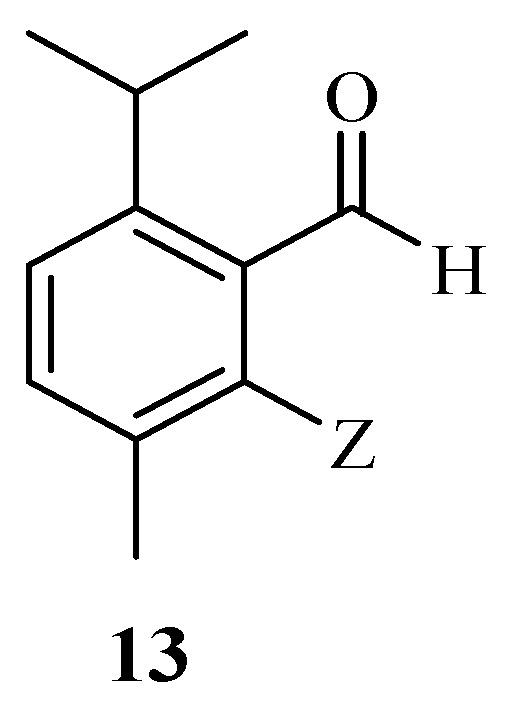
Chemical structure of carvacrol–aldehyde derivative, **13**.

The in silico pharmacokinetic and pharmacodynamic studies of the carvacrol–aldehyde hybrid derivative **13** (Figure 15) reported by Bansal et al. displayed an anti-metastatic effect [56]. The binding affinity energy of the hybrid drug was −5.3 kcal/mol with a good interaction with metastasis-associated protein 1. It also displayed druglikeness properties according to Lipinski’s rule of five. Therefore, further studies, such as in vitro and in vivo studies, are recommended for this potent anticancer compound [56].

**Figure 16:**
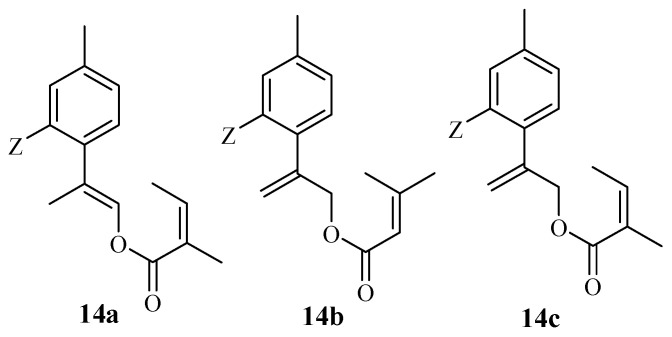
Chemical structures of thymol hybrid derivatives **14a**–**c** modified by Yu et al.

Five thymol hybrids were synthesized by Yu et al., and their anticancer efficacy was evaluated on different human cancer cells: Hep G2, A549, MCF-7, and HeLa [57]. Against these human cancer cell lines, hybrid **14a**–**c** (modified on the isopropyl side of thymol) (Figure 16) displayed significant activity (i.e., IC_50_ values in the range of 6.24–11.96 µM) which was comparable to that of cisplatin (IC_50_ values in the range of 6.20–10.95 µM). These findings reveal that the anticancer efficacy of thymol can be enhanced via modifications, promoting its application in the design of anticancer drugs [57].

**Figure 17:**
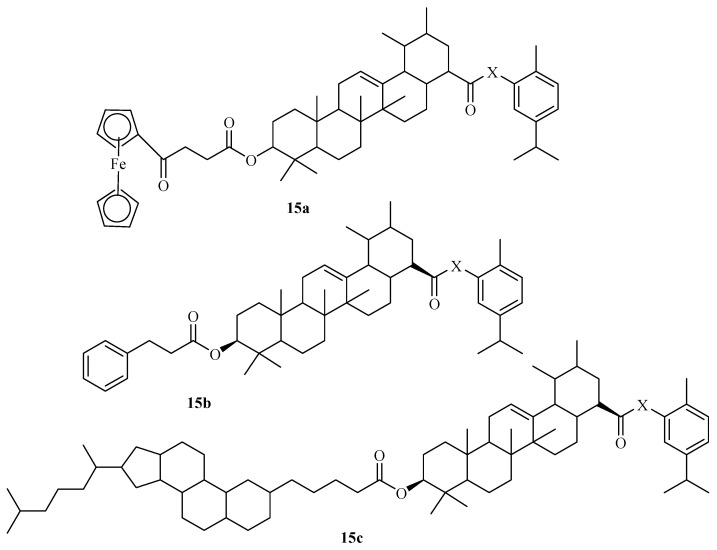
Chemical structures of ursolic–carvacrol hybrid drugs **15a**–**c**.

Khwaza et al. synthesized ursolic–carvacrol derivatives **15a**–**c** (Figure 17) and evaluated them against three cancer cells, including MCF-7, MD-MBA-231, and HeLa [58]. The synthesized compounds displayed inferior anticancer results with IC_50_ values in the range of 51.05–64.75 μg/mL when compared to 49 μg/mL of ursolic acid. Modifying the hydroxyl group on the carvacrol moiety and the di-substitution of the hydroxyl and carboxylic groups of the ursolic acid moiety compromised the anticancer activity of the compounds [58]. Therefore, further structural elucidation is recommended. Summary of the anticancer activity, SAR, and mechanism of action of carvacrol and thymol hybrids (Table 2).

## 4. Carvacrol/Thymol Hybrid Compounds with Antibacterial Activity

The antibacterial effects of thymol and carvacrol include microbes encased in biofilms. Their derivatives have drawn a lot of interest owing to their antibacterial, anti-HIV, antifungal, and antiviral properties [59,60]. The evaluation of the antibacterial activity of derivatives of thymol and carvacrol against a variety of bacterial strains revealed the impact of structural modifications [61]. Since thymol and carvacrol consist of hydroxyl groups and are structurally isomeric, they both exhibited comparable levels of inhibitory effects [62].

**Figure 18:**
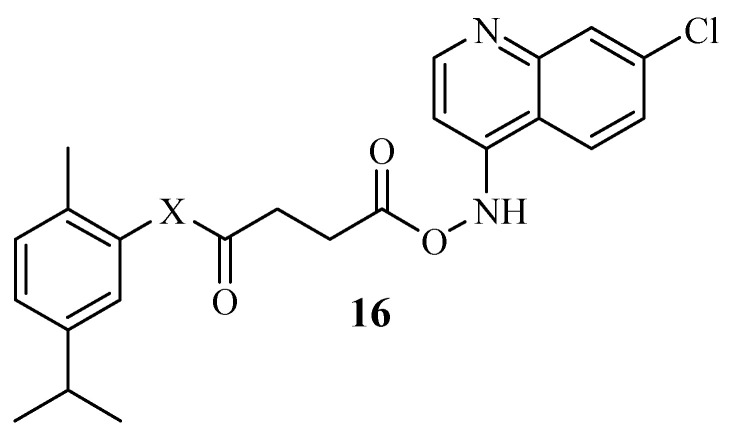
Hybrid **16** synthesized by Mbese et al.

Mbese et al. synthesized carvacrol ester hybrids (Figure 18) and tested them against different bacterial strains [51]. The hybrids displayed good antibacterial activity with minimum inhibitory concentration (MIC) values in the range of 1.25–3.3 μg/mL. However, they did not induce significant antibacterial effects when compared to the parent drug (carvacrol). Moreover, compound **16** was the most active compound with MIC values in the range of 0.10–0.68 μg/mL [51]. The 4-aminoquinoline scaffold was influential in the antibacterial activity of this compound. Additionally, the modification of the hydroxyl group resulted in the compromised antibacterial activity of the hybrids. Thus, collaborating findings were reported by Ranjbar-Karimi Alireza [63]. Therefore, further studies of these carvacrol–ester hybrids are essential [51].

Thymol hybrid compounds **12a**–**c** (shown in Figure 14) displayed improved antibacterial effects which were comparable to those of their parent drug, thymol, against E. coli with MIC values between 10.66 and 11 µM, as reported by Sahin et al. [55]. Compound **12c** with two halogens was reported as the most effective drug among the synthesized hybrid drugs against the aforementioned bacterial strain. The introduction of halogens to the side chain of the benzylideneamino moiety influenced the antibacterial activity of compound **12c**. Therefore, thymol hybrids are a promising lead in a new generation of antibacterial drugs [55].

Khwaza et al. synthesized several hybrid drugs (Figure 17) and evaluated their antibacterial activity against selected strains of bacteria [58]. Among the synthesized hybrids, **15a** and **15b** carvacrol hybrid drugs displayed remarkable antibacterial activities with MIC values of 15.63 μg/mL against Proteus vulgaris and Proteus mirabilis. Additionally, these compounds displayed comparable antibacterial activities to those of ursolic acid [58]. Ghod Elahi et al. documented that modifying the carvacrol moiety can improve the antibacterial effect [64]. Modifying carvacrol with a peptide improved its antibacterial activity against several bacteria strains, including Pseudomonas aeruginosa and Staphylococcus epidermidis, revealing promising antibacterial activity with MIC values in the range of 0.5–1 μg/mL. However, further studies are recommended [64].

**Figure 19:**
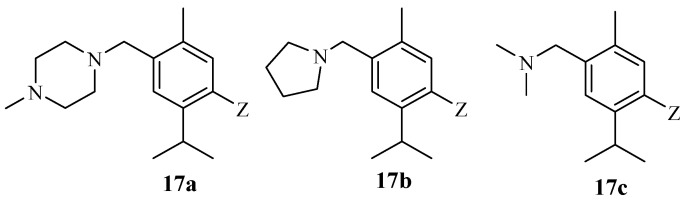
Chemical structures of thymol–cyclic amine hybrid compounds **17a**–**c**.

Kumar et al. documented the antibacterial activity of thymol hybrid derivatives synthesized through a combination of thymol, cyclic amines, and sulfanilamides [65]. The hybrids were evaluated against several bacterial strains, including S. aureus and E. coli. Hybrids **17a**–**c** with a cyclic amine moiety (Figure 19) displayed promising antibacterial effects against S. aureus and E. coli. Hybrids **17a** and **17b** showed MIC values of 12.5 μg/mL and 3.12 μg/mL, respectively, with hybrid **17c** displaying an MIC value of 6.25 μg/mL against S. aureus and E. coli. The presence of the aminomethyl group was responsible for the improved antibacterial activity of these compounds. These findings suggest that thymol derivatives are potent antibacterial agents [65].

**Figure 20:**
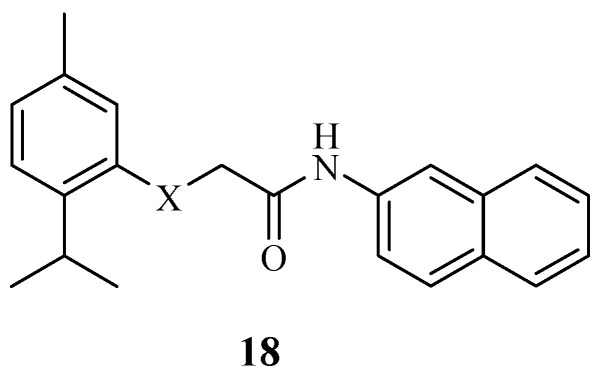
Chemical structure of thymol ether hybrid derivative **18**.

Patil and Pawar synthesized various thymol ether hybrids and evaluated them against four different antibacterial strains [66]. Hybrid **18** (Figure 20) displayed a high antibacterial activity with a 5 mm zone of inhibition against P. valgaries, S. aureus, and B. subtilis. However, this compound showed no significant effect against E. coli. Although the hybrids were selective towards the antibacterial strains, the introduction of the thymol moiety was responsible for the improved activity [66,67].

**Figure 21:**
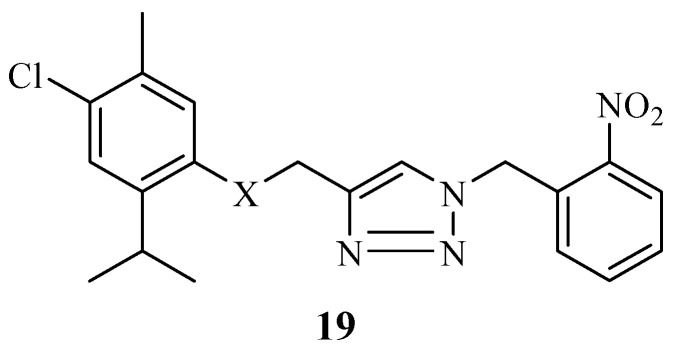
Chemical structure of 1,2,3-triazole-thymol hybrid derivative **19**.

1,2,3-triazole-thymol hybrid derivatives were synthesized by Addo et al. after they displayed promising antibacterial results when evaluated against several bacterial strains [68]. Most of the synthesized compounds displayed a similar or superior antibacterial activity comparable to that of ampicillin, the control, depending on the bacterial strains used. Antibacterial studies of the compound without chlorine on the fourth position of the thymol moiety against K. pneumonia revealed that the bacterial strain developed resistance. Thus, replacing hydrogen with halogen improved the antibacterial activity of the compounds. Moreover, compound **19** (Figure 21) with a mean zone inhibition of 24.7 mm was the most active antibacterial compound among its counterparts, and it was also comparable to the control drug with a 30 mm mean zone of inhibition [68].

**Figure 22:**
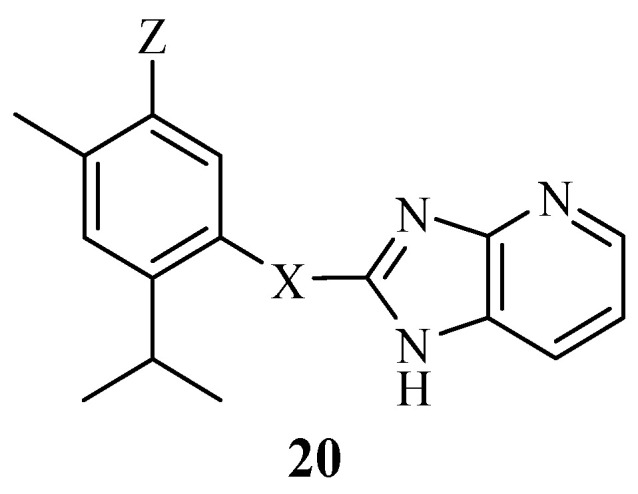
Chemical structure of benzimidazole-carvacrol hybrid derivative **20**.

Bhoi et al. synthesized nine benzimidazole–carvacrol hybrids and evaluated them against four different bacterial strains, such as *E. coli, S. aureus, S. pyogenus*, and *P. aeruginosa* [69]. The hybrids displayed remarkable antibacterial activities against all four strains compared to those of some of the control drugs that were used. However, they were selective towards the bacterial strains with MIC values between 12.5 and 250 µg/mL. Hybrid **20** (Figure 22) was the most active antibacterial compound with MIC values of 12.5 µg/mL (*S. aureus*), 25 µg/mL (*E. coli*), 50 µg/mL (*S. pyogenus*), and 25 µg/mL (*P. aeruginosa*), respectively [69]. The position and the nature of the substituent, e.g., the introduction of fluoroalkyl and alkyl groups on the benzimidazole moiety, influenced the antibacterial activity of the hybrids. The trend was inconsistent and depended on the type of bacterial strain used. Hybridizing natural products with azoles and the structural modification of the hybrids can result in effective antibacterial agents that can overcome resistant bacterial strains [68,69,70,71]. Summary of antibacterial activity, SAR, and mechanism of action of carvacrol and thymol hybrids is shown in Table 3.

## 5. Conclusions and Future Strategies

The development of new and effective antibacterial and anticancer therapeutics is urgent. Thus, the use of plant-based bioactive molecules to develop new drugs is a promising approach, as compounds extracted from traditional plants have some good features, such as reduced toxicity levels and several biological activities. Drug resistance has been a major issue in the treatment of various diseases, including cancer and bacterial infections. Thus, the number of cases and deaths is increasing due to drug resistance issues. The socio-economic burden as a result of cancer disease and bacterial infections has been projected to be a major problem in the near future. Hence, exploring sustainable strategies for drug development is crucial.

Carvacrol and thymol possess several biological activities. Hybrid drugs are promising therapeutics with unique pharmacological features, making them effective drug molecules. These two isomeric compounds are promising moieties that can be hybridized with other pharmacophores to develop antibacterial and anticancer agents with limited challenges. Some research reports have shown that the antibacterial and antioxidant activities of these isomeric compounds are attributed to their free hydroxyl group [29,30]. Hence, its modification must be avoided [51,63].

However, the site of modification for these drugs is still debatable, as most researchers are using the hydroxyl group as a site of modification. Moreover, most of the promising hybrids reported in this review were combined via the hydroxyl group to form ester linkers, and they displayed potential anticancer and antibacterial effects. Hence, in vivo studies of these compounds are crucial to validate the biological activities and the modes of action of the reported compounds. The reported compounds were selective towards some cancer cell lines and bacterial strains. Thus, the structural activity relationship trends are not consistent. However, in some compounds, the site of the substitution and the nature of the substituent influenced the biological activities of these drugs. Hence, more studies must be performed on these hybrids with more structural modifications using a wider range of cancer cell lines and bacterial strains, as well as in vivo evaluations. Although hybrid compounds are a promising and interesting approach to developing novel and effective therapeutic agents, some structural modifications have resulted in ineffective compounds. For instance, most of the hybrid compounds were characterized by high molecular weights, which sometimes violate Veber’s and Lipinski’s rule [21,72]. Thus, antibiotic hybrid drugs with molecular weights of more than 600 Da are a major concern for gram-positive bacteria, due to the possibility of non-cellular uptake through the dual membrane of the bacteria. Therefore, the molecular weights of the hybrid compounds must not be overlooked [21,72]. The modes of action of the parent drugs can be lost if the structural modifications are performed on functional groups responsible for the parent drug’s mechanism of action. Although hybrid compounds are dual-targeting drugs, hybrids can bind to one original binding site of the parent drug with the possibility of not reaching other targeting sites [72]. Thus, the type of linker and the site of attachment should be considered when developing hybrid compounds.

## Figures and Tables

**Table 1:** Mechanism of action and limitations of carvacrol and thymol.

Carvacrol and Thymol	Antibacterial	Anticancer
Mechanism of action	Cell membrane interruption [37] Inhibition of efflux pump Inhibition of membrane-bound ATPase [38] Inducing permeability [38]	Apoptosis induction [39] Production of reactive oxygen species (ROS) [27] Alteration of mitochondrial membrane [40] Cell growth inhibition [40]
Limitations	Drug resistance Increased toxicity Poor water solubility Low stability [41,42]	Drug resistance Increased toxicity Poor water solubility [42] High hydrophobicity [41]

**Table 2:** Summary of the anticancer activity, SAR, and mechanism of action of carvacrol and thymol hybrids.

Hybrid	Type of Cancer Cells Active Against	SAR	Mode of Action	Reference
**3**	MCF-7	The introduction of the benzene ring, sulfonamide group, and halogens influenced the anticancer activity.	Promote apoptosis	[1]
**4**	HT-1080	Replaced the hydrogen with a methyl group improved the anticancer effect.	Induce early and late apoptosis	[9]
**5a**–**b**	A-549/HT-1080	The ether group on the thymol moiety was influential on the improved activity.	-	[18]
**6a**–**m**	HCT-116/MCF-7/HepG2	The number and position of substituents influenced the anticancer activity.	-	[48]
**7a**–**b**	-	No noticeable SAR trend.	-	[50]
**8**	MCF-7/MCF-12A	The anticancer improvement was attributed to the use of the ester linker.	-	[51]
**10a**–**c**	SH-SY5Y/HEK-293	The type of halogen and the position of nitro group influenced the cytotoxic effect.	-	[53]
**11a**–**b**	MCF-7/HT-29	No significant trend.	Induce apoptosis	[54]
**12a**–**c**	PC3/DLD-1	SAR displayed no significant trend. However, the introduction of halogens compromised the activity.	-	[55]
**14a**–**c**	Hep G2/A549/MCF-7/HeLa	Modification of isopropyl side of thymol via ester linkers promoted their anticancer activity.	-	[57]
**15a**–**c**	MCF-7, MD/MBA-231/HeLa	Destruction of hydroxyl group compromised their anticancer activity.	-	[58]

**Table 3:** Summary of antibacterial activity, SAR, and mechanism of action of carvacrol and thymol hybrids.

Hybrid	Bacterial Pathogens Active Against:	SAR	Reference
**12a**–**c**	*E. coli*	The introduction of halogens influenced the antibacterial activity of these compounds.	[55]
**15a**–**b**	*Proteus vulgaris/Proteus mirabilis*	Hybridizing carvacrol and ursolic acid via an ester linker improved their antibacterial activity.	[58]
**16**	*E.coli/S. aureus*	Modification of the hydroxyl group of carvacrol moiety resulted in compromised antibacterial activity.	[51,63]
**17a**–**c**	*S. aureus/E.coli*	The introduction of cyclic amine moiety with aminomethyl groups into thymol improved the antibacterial activity.	[65]
**18**	*P. valgaries/S. aureus/B.subtilis*	The introduction of thymol moiety was responsible for the improved activity.	[66,67]
**19**	*K. pneumonia*	Replacing hydrogen with halogen improved the antibacterial activity of the compounds.	[68]
**20**	*E. coli/S. aureus/S. pyogenus/P. aeruginosa*	The introduction of fluoroalkyl and alkyl groups on benzimidazole moiety influenced the antibacterial activity of the hybrids.	[69]

## Data Availability

Not applicable.
